# Paracervical block in office hysteroscopy with vaginoscopic approach to reduce patients’ pain perception: a propensity score matching analysis

**DOI:** 10.1080/07853890.2026.2688466

**Published:** 2026-06-24

**Authors:** Davide De Santo, Roberta Marie Gentile, Giuseppe Mirenda, Giovanni Di Lorenzo, Maria Sole Scalia, Chiara Dal Pozzolo, Elena Magni, Federica Scrimin, Alessandro Arena, Paolo Casadio, Federico Romano, Giuseppe Ricci

**Affiliations:** ^a^ Institute for Maternal and Child Health, IRCCS “Burlo Garofolo”, Trieste, Italy; ^b^ Department of Medical, Surgical and Health Sciences, University of Trieste, Trieste, Italy; ^c^ Clinical Epidemiology and Public Health Research Unit, Institute for Maternal and Child Health-IRCCS “Burlo Garofolo”, Trieste, Italy; ^d^ Division of Gynecology and Human Reproduction Physiopathology, IRCCS Azienda Ospedaliero Universitaria, Bologna, Italy; ^e^ Department of Medical and Surgical Sciences, DIMEC, University of Bologna, Bologna, Italy

**Keywords:** Paracervical block, hysteroscopy, office hysteroscopy, pain management

## Abstract

**Background:**

Pain perception is a major limitation of office hysteroscopy and may lead to premature suspension of the procedure. Current guidelines do not routinely recommend local analgesia, and the effectiveness of paracervical block during office hysteroscopy performed with a vaginoscopic approach remains insufficiently investigated.

**Objectives:**

The primary objective was to assess whether paracervical block is associated with reduced pain perception during office hysteroscopy with a vaginoscopic approach. The secondary objective was to evaluate its impact on procedure suspension.

**Materials and Methods:**

his retrospective observational cohort study included patients undergoing diagnostic and/or operative office hysteroscopy between January 2018 and January 2022. Paracervical block was administered without speculum or tenaculum. Confounders were addressed using multivariable regression and propensity score matching.

**Results:**

A total of 2,028 patients were analyzed. In patients who did not receive PB, nulliparity was associated with severe pain (aOR 2.87, 95% CI 1.20–6.85) and procedure suspension (aOR 3.07, 95% CI 1.27–7.39), and cervical canal stenosis with markedly increased odds of both severe pain (aOR 23.17, 95% CI 10.44–51.40) and suspension (aOR 25.10, 95% CI 11.19–56.30). In patients receiving PB, nulliparity was no longer significantly associated with either outcome, and the effect of cervical canal stenosis was substantially attenuated (aOR 2.68, 95% CI 1.57–4.58 for severe pain; aOR 3.70, 95% CI 2.07–6.62 for suspension).

**Conclusion:**

Overall, in this large retrospective cohort, paracervical block performed through a vaginoscopic approach was associated with reduced pain perception and procedure suspension in selected patients.

## KEY MESSAGES

- In a large retrospective cohort, paracervical block performed *via* a vaginoscopic approach was associated with reduced pain perception during office hysteroscopy.
- The use of paracervical block was associated with substantially lower odds of procedure suspension, particularly in nulliparous women and in patients with cervical canal stenosis.
- Paracervical block may be considered as part of a structured pain-management strategy in patients with anticipated difficult cervical access, pending confirmation by prospective randomized trials.

## Introduction

Hysteroscopy is the “gold standard” technique in the diagnosis and management of intracavitary uterine pathology [1]. With the introduction of smaller hysteroscopic instruments, office hysteroscopic procedures have evolved to favor a “see and treat” approach [2], allowing several operative procedures, previously limited to the operating room (OR), to be performed in the office setting. Office hysteroscopy (OH), mainly thanks to the vaginoscopic approach (VA), which avoids the use of the speculum and tenaculum, allows a rapid recovery and a threefold reduction of the estimated costs with respect to the OR [3]. OH has a success rate of 77–97.2%, with an estimated failure rate of approximately 10%. The main reasons for suspension of the procedure include pain perceived by the patient in 29.4%, stenosis of the cervical canal in 28.2%, poor visibility during the procedure in 20.8% and vagal syndrome in 0.5–9% of cases [4,5]. The primary cause of pain is the manipulation of the cervix during the passage of the hysteroscope through the cervical canal (CC) [5]. Even with VA, the pain associated with the procedure has been reduced but not eliminated [6]. Pain perception during OH is multifactorial, with uterine distension, operative steps, operator experience and patient anxiety contributing alongside cervical manipulation [5,7]; any single analgesic intervention should therefore be interpreted within this broader framework.

Current guidelines do not reach a consensus on pain management during hysteroscopy, and historically the main international documents have advised against the routine use of paracervical block (PB) during OH [8–10]. Importantly, however, these recommendations are based almost entirely on studies in which PB was performed with speculum and tenaculum, an inherently more invasive technique that is itself a source of pain and vasovagal stimulation. Consequently, the role of PB administered without speculum and tenaculum, in the context of a pure vaginoscopic approach, has been only marginally investigated.

More recently, the ACOG Clinical Consensus on Pain Management for In-Office Uterine and Cervical Procedures has broadened the analgesic options that should be discussed with patients undergoing office hysteroscopy, explicitly including paracervical block, and has renewed the clinical relevance of evaluating PB in a modern vaginoscopic setting [11].

In our institution, PB delivered *via* the vaginoscopic route with a dedicated Vincenti’s needle has become part of the standard pain-management strategy for OH; the present study asks whether, within a fully vaginoscopic workflow, it provides an additional margin of comfort and procedural success in patients in whom VA alone is anticipated to be insufficient.

The aim of the study was to evaluate the contribution of PB during OH performed with VA, without the use of speculum and tenaculum, with a particular focus on patients at higher risk of severe pain or procedure failure.

## Materials and methods

This is a retrospective observational cohort study conducted on a consecutive series of patients undergoing diagnostic and/or operative OH performed at the Hysteroscopy and Day Surgery service in the S.C.U. Obstetric and Gynecological Clinic of IRCCS Burlo Garofolo, Trieste, from January 2018 to January 2022. Written informed consent was obtained from all patients for the use of their clinical data for research purposes.

The primary outcome was to evaluate whether PB administered during VA was associated with a reduction in perceived pain.

Secondary outcome was to evaluate the association between PB and the rate of procedure suspension during OH.

A dedicated nursing team collected the patient’s vital parameters and assessed perceived pain using the Numeric Rating Scale (NRS) [12], before, during, and after the procedure. A score of 0 is considered no pain, 1–3 mild pain, 4–6 moderate pain, 7–10 severe pain [13]. The duration of the examination, the need for painkillers, and the side effects were recorded, and the assessment of discharge criteria was performed using the Modified Post-Anesthetic Discharge Scoring System (MPADSS) scale [14].

The cases were extracted from clinical records, and a specific anonymous database was created.

Exclusion criteria included missing anamnestic data, incomplete hysteroscopic evaluation, absence of the pain assessment form, missing vital parameters, or lack of documentation on drugs administered.

This study was approved by the local IRB (IRB-Burlo 01/2022, 09.02.2022) and performed according to the ethical standards in the Declaration of Helsinki.

## Office hysteroscopic procedure

All procedures performed included diagnostic or operative hysteroscopies in an office setting, according to the classification proposed by the International Consensus Statement for Recommended Terminology Describing Hysteroscopic Procedures [15].

The procedure was standardized for all patients and divided into the following steps. The patients were placed in lithotomy position. No premedication or pre-analgesia was provided prior to the examination [16]. The hysteroscopy was performed with a vaginoscopic approach [6,16]; no speculum and tenaculum to grasp the cervix was ever used. No dilation of the CC was performed. Saline solution was used as the irrigation fluid, delivered by an automatic pump system (ENDOMAT Select KARL STORZ SE & Co. KG, Tuttlingen, Germany) at a constant working pressure of 60 mmHg.

The following 5-mm hysteroscopes were used:

- “Bettocchi Hysteroscope” (KARL STORZ SE & Co. KG, Tuttlingen, Germany), with HOPKINS optical system with 30° oblique vision, equipped with an operative canal to use 5 Fr. semirigid surgical instruments and bipolar electrode;
- As of 2019, the 15 Fr. Bipolar Resectoscope (KARL STORZ SE & Co. KG, Tuttlingen, Germany) was introduced in our setting.

The procedure was suspended in case of severe pain perception, adverse side effects onset or if the procedure was considered not feasible in office setting.

After the procedure, patients were monitored for ten minutes in a dedicated waiting room and then discharged.

The procedures were carried out by different operators with heterogenous expertise, making the data closer to real clinical experience.

### The paracervical block

**Figure 1.**
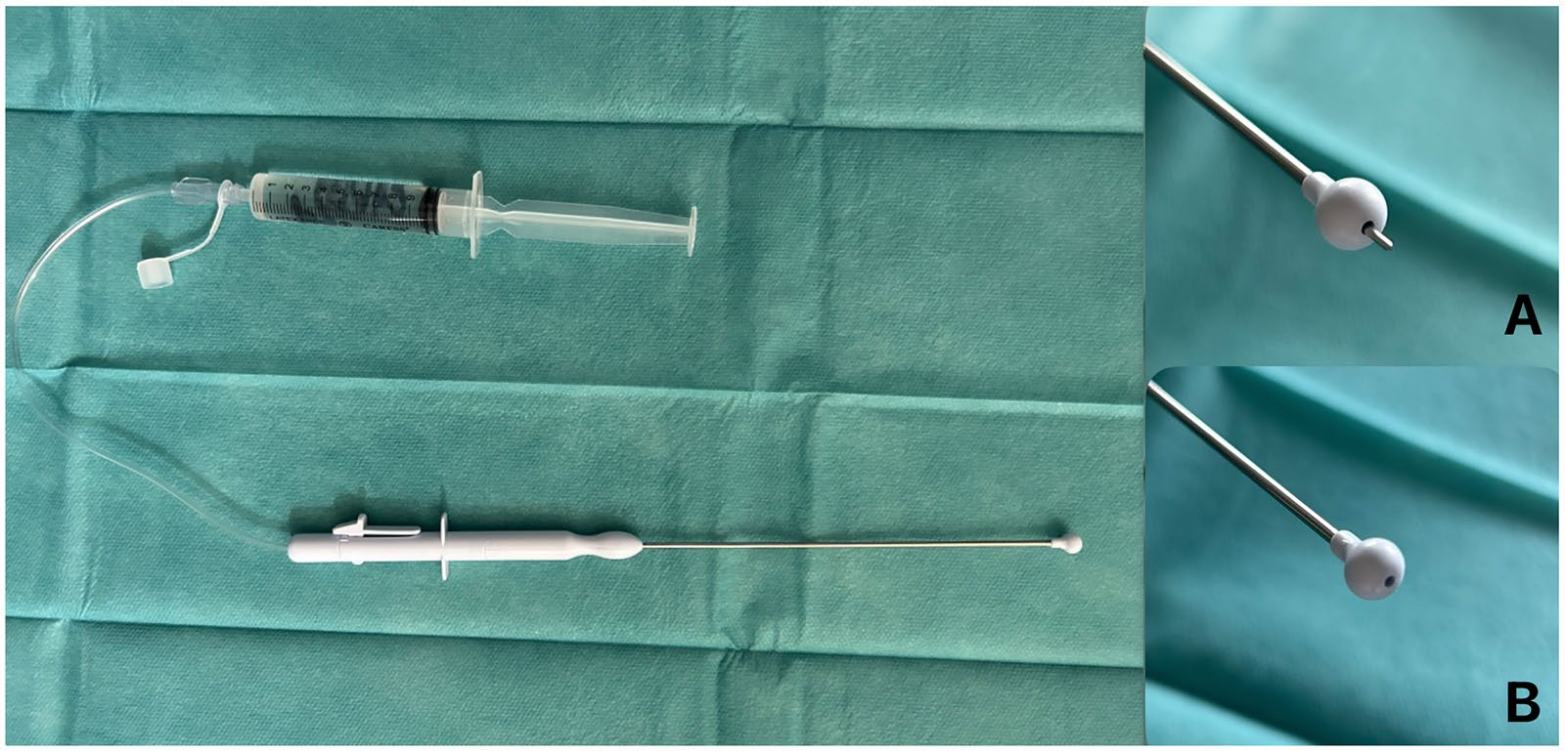
Vincenti’s needle (Bioengineering Laboratories Spa *via* Isonzo 9/B - 20036 Meda - MB). A: armed; B: closed.

**Figure 2.**
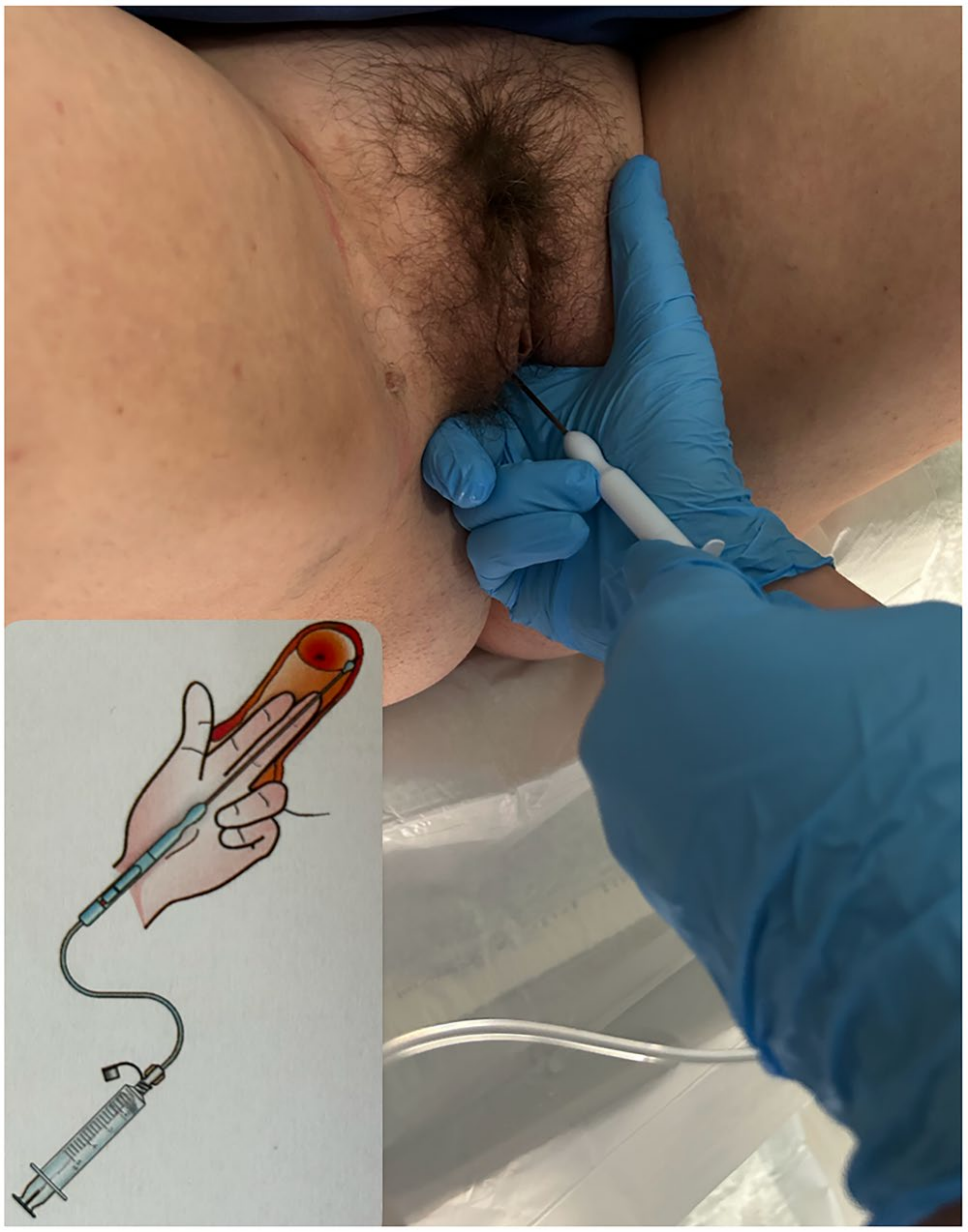
execution of PB using the “Vincenti’s” needle (Bioengineering Laboratories Spa *via* Isonzo 9/B - 20036 Meda - MB), without using speculum and/or tenaculum.

When performed, PB was administered without the need for speculum and tenaculum. After genital disinfection with a sodium hypochlorite-based solution, the cervix was identified by single-digit vaginal examination. A special needle called “Vincenti’s needle” (Bioengineering Laboratories Spa *via* Isonzo 9/B-20036 Meda - MB) was introduced directly into the vagina (Figures 1, 2).

The local anesthetic was administered by injecting 5 mL, per site, of Mepivacaine Hydrochloride 20 mg/mL into the postero-lateral vaginal fornix, bilaterally. In this way, the drug reaches the utero-vaginal plexus and at the base of the broad ligament, close to the lateral parametrium, theoretically inhibiting nociception of the CC and of the body of the uterus [17,18].

The time of injection can be estimated at a few seconds per site. The hysteroscopic procedure was delayed for an average of 2–3 min to allow the drug diffusion. During this time, the hysteroscopic instruments are correctly prepared. If needed, a transvaginal ultrasound was performed to assess the pre-operative scenario and to aid hysteroscopy.

Pain perception during the PB administration following this technique is usually absent.

The decision to administer PB was operator-dependent and based on each operator’s clinical judgement, integrating prior experience with patients’ pain perception and the anticipated technical complexity of the procedure. This pragmatic, indication-based approach reflects real-world office practice and provided the empirical basis for the practice-oriented framework subsequently developed from these data.

### Statistical analysis

The statistical analysis of the data was conducted by the Clinical Epidemiology and Health Services Research Unit of IRCCS Burlo Garofolo.

Data were reported as numbers and percentages for categorical variables, and the Chi-square test or Fisher’s exact test, when appropriate, were used for group comparison (PB vs no PB, perception of severe pain vs no pain or mild pain, suspended vs non-suspended procedure). Continuous variables were reported as median and interquartile range (IQR), and the independence of distribution between groups was assessed using the Mann-Whitney test. Successively, separate multiple logistic models were estimated for patients who received PB (cases) and patients who did not receive PB (controls) to study the potential influence of PB on selected candidate risk factors for the outcomes of interest (suspension of the procedure and severe pain perception). The choice of estimating separate models was made to provide results easier to read and interpret. Before model estimation, Propensity Score Matching (PSM) was performed to make the two groups (cases and controls) homogeneous with respect to unbalanced demographic and clinical characteristics: age, childbirth, and comorbidity. These factors were the primary a priori criteria for administering PB. A general logistic model with interaction terms was finally estimated to evaluate the hypotheses made on the previous models. All statistical analysis was performed using R Software, Version 4.1.1 (R Foundation for Statistical Computing, Vienna).

## Results

**Figure 3.**
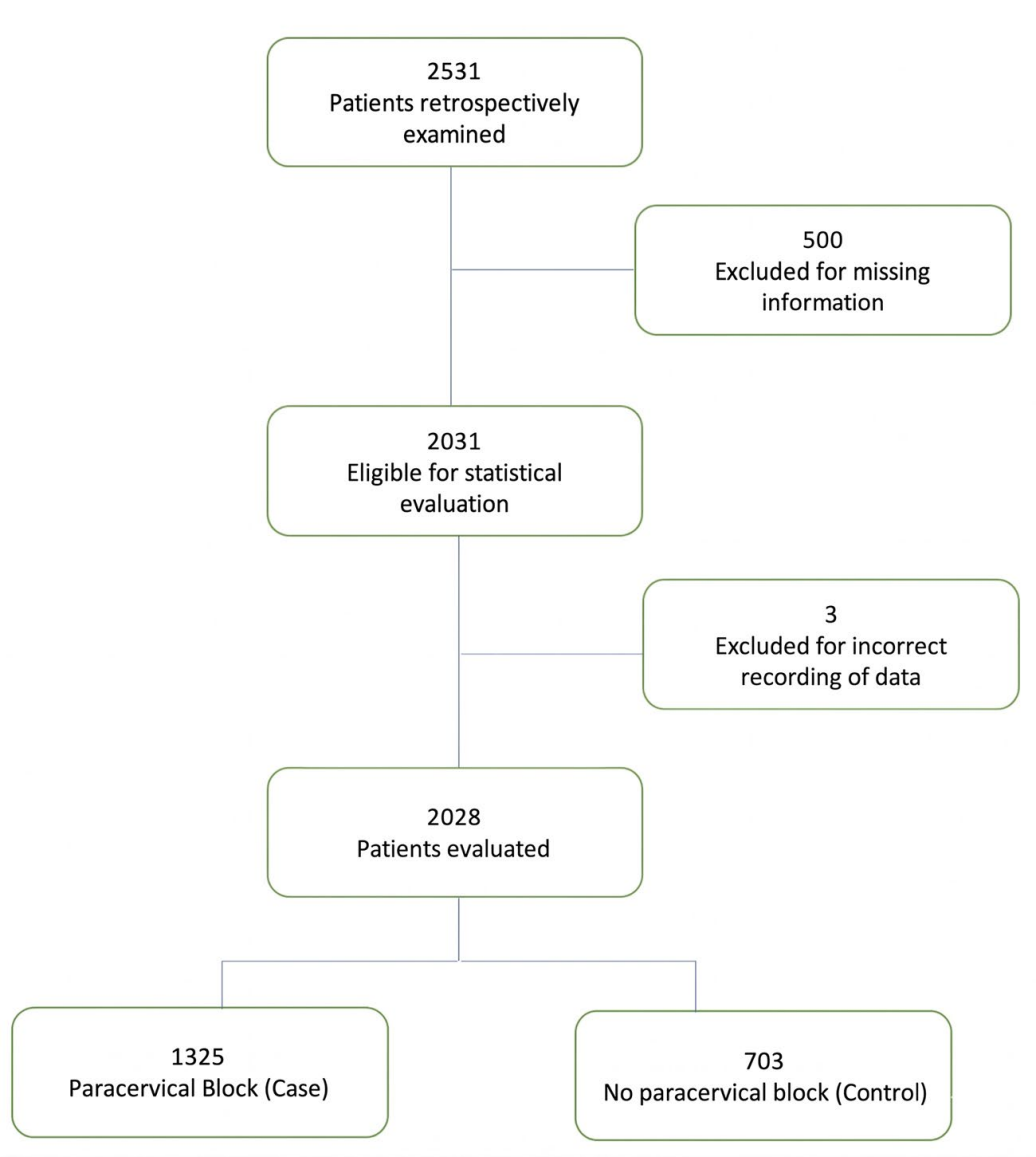
Study design.

A total of 2,531 patients were eligible for the study, and of these, 503 were excluded according to exclusion criteria: 500 for missing information and three patients for incorrect recording of data. The remaining 2,028 patients were included in the study (Figure 3). The clinical characteristics of the patients are presented in Table 1.

**Table 1.** Patients characteristics according to the use of paracervical block (PB).

	All patients	No PB	PB	
	*n* = 2028	*n* = 703	*n* = 1325	*p*-value
Clinical characteristics				
Age, years*	51 (43–60)	50 (43–58)	51 (43–61)	**0.03**
Childbirth**				**<0.001**
Nulliparous	671 (33.1)	166 (23.6)	505 (38.1)	
No vaginal delivery	235 (11.6)	70 (10.0)	165 (12.5)	
Vaginal delivery	1122 (55.3)	467 (66.4)	655 (49.4)	
Menopause**	1001 (49.5)	345 (49.4)	656 (49.6)	0.95
Weight, kg*	65 (58–77)	66 (58–78)	65 (58–77)	0.49
BMI*	24 (21–28)	24 (21–28)	24 (21–28)	0.92
Diseases Y/N**	866 (42.7)	262 (37.3)	604 (45.6)	**<0.001**
Cardiovascular**	311 (15.3)	85 (12.1)	226 (17.1)	**<0.01**
Endocrinological**	428 (21.1)	133 (18.9)	295 (22.3)	0.08
Oncological**	168 (8.3)	49 (7.0)	119 (9.0)	0.12
Genitourinary system**	100 (4.9)	27 (3.8)	73 (5.5)	0.10
Other**	140 (6.9)	42 (6.0)	98 (7.4)	0.23
Indications				
Polyp**	587 (28.9)	200 (28.4)	387 (29.2)	0.72
AUB**	593 (29.2)	186 (26.5)	407 (30.7)	**0.04**
Endometrial thickening**	269 (13.3)	84 (11.9)	185 (14.0)	0.20
Myoma**	130 (6.4)	54 (7.7)	76 (5.7)	0.09
Menometrorrhagia**	327 (16.1)	138 (19.6)	189 (14.3)	**<0.01**
Uterine anomalies**	41 (2.0)	8 (1.1)	33 (2.5)	**0.04**
Sterility**	196 (9.7)	75 (10.7)	121 (9.1)	0.27
Incarcerated IUD**	38 (1.9)	21 (3.0)	17 (1.3)	**0.01**
Other**	229 (11.3)	82 (11.7)	147 (11.1)	0.70
Findings				
Endometrial polyp**	728 (35.9)	240 (34.1)	488 (36.8)	0.23
Cervical polyp**	204 (10.1)	62 (8.8)	142 (10.7)	0.18
Myoma**	235 (11.6)	75 (10.7)	160 (12.1)	0.35
Endometrial hyperplasia**	416 (20.5)	123 (17.5)	293 (22.1)	**0.01**
Suspect of cancer**	88 (4.3)	24 (3.4)	64 (4.8)	0.14
Uterine anomalies**	117 (5.8)	26 (3.7)	91 (6.9)	**<0.01**
Stenosis**	343 (16.9)	92 (13.1)	251 (18.9)	**<0.01**
Synechiae**	214 (10.5)	60 (8.5)	154 (11.6)	**0.03**
Dysfunctional endometrium**	234 (11.5)	85 (12.1)	149 (11.3)	0.57
Pyometra/Mucometra**	36 (1.8)	3 (0.4)	33 (2.5)	**<0.01**
Other**	139 (6.9)	41 (5.8)	98 (7.4)	0.19
No pathological findings**	284 (14.0)	125 (17.8)	159 (12.0)	**<0.001**
Procedure				
Diagnostic/Biopsy**	1671 (82.4)	574 (81.7)	1097 (82.8)	0.52
Polypectomy**	552 (27.2)	170 (24.2)	382 (28.8)	**0.03**
Myomectomy**	16 (0.8)	3 (0.4)	13 (1.0)	0.29
Metroplasty**	39 (1.9)	10 (1.4)	29 (2.2)	0.23
Synechiae lysis**	59 (2.9)	12 (1.7)	47 (3.5)	**0.02**
IUD removal/insertion**	47 (2.3)	25 (3.6)	22 (1.7)	**<0.01**
Abortive material retention**	13 (0.6)	4 (0.6)	9 (0.7)	1.0
Procedure length, min*	15 (10–20)	15 (10–20)	15 (10–25)	**<0.001**
Devices used				
15 Fr bipolar resector**	35 (1.75)	9 (1.3)	26 (2.0)	0.262
Bipolar mini electrode**	100 (4.9)	30 (4.3)	70 (5.3)	0.315

* Median and IQR.

** “Yes” number and percentage.

The median age of the population was 51 years (IQR 43–60). Nulliparity was recorded in 671 patients (33.1%), while 1,357 (66.9%), were multiparous. Vaginal delivery occurred in 1,122 (55.3%) cases. The median BMI was 24 (IQR 21–28). 42.7% of patients had comorbidities at the time of the examination (Table 1): 428 metabolic and/or endocrine (21.1%), 311 cardiovascular (15.3%), 168 oncologic (8.3%), and 140 patients (6.9%) had other pathologies (Table 1).

Comparing the general information of the population in study, we found that PB was administered differently depending on age (higher for patients who received PB (51 years, IQR 43–61) than controls (50 years, IQR 43–58; *p* = 0.03), and vaginal delivery (lower proportion of PB administration in patients with history of VD, 66.4% controls vs 49.4% cases; *p* < 0.001). Patients with comorbidities were more likely to receive PB (45.6 vs 37.3%; *p* < 0.001).

The most common reason for hysteroscopic evaluation was post-menopausal abnormal uterine bleeding (AUB) in 593 patients (29.2%), followed by suspected endometrial polyps in 587 patients (28.9%) and menometrorrhagia unresponsive to medical therapy in 327 patients (16.1%). Unexplained sterility was investigated with hysteroscopy in 196 (9.7%) patients (Table 1). Endometrial polyps were diagnosed in 728 patients (35.9%), in 204 cases (10.1%) the polyp was found in the CC. Endometrial hyperplasia was found in 416 patients (20.5%). Histologically confirmed endometrial cancer was detected in 88 patients (4.3%). Uterine malformations were reported in 117 cases (5.8%). The presence of synechiae was described in 214 (10.5%) patients. Tight stenosis of the CC was found in 343 patients (16.9%).

In 284 (14%) cases, no pathological finding was evident, and these patients were less likely to receive PB (17.8 vs 12%, *p* < 0.001). Endometrial hyperplasia, uterine abnormalities, CC stenosis, synechiae and pyometra/mucometra were more common among patients who received PB compared to controls (Table 1).

Polypectomy was performed in 552 cases (27.2%). Endometrial biopsy was the most performed procedure in 1671 cases (82.4%). Polypectomy and lysis of synechiae were associated with the use of PB (Table 1). The 15 Fr bipolar resector was used for polypectomy or myomectomy (G0 myomas) in 35 patients. The bipolar minielectrode was used in 100 cases (4.9%) (Table 1).

Furthermore, the median procedure length (calculated from the start of the hysteroscopic procedure) was 15 min (IQR 10–20) and PB was significantly associated with longer procedures (*p* < 0.001; Table 1).

PSM was used to make the population homogeneous for demographic characteristics: age, childbirth, and comorbidity (Table 2), considering these variables the only a priori criteria for the examined population.

**Table 2.** aOR Of paracervical block use after propensity score matching (PSM).

Paracervical block	aOR	[95% IC]	*p*-value
Age	1.01	[1.00 –1.01]	0.143
No vaginal delivery	0.94	[0.77–1.14]	0.547
Diseases	1.05	[0.86 –1.27]	0.632

Stratified analyses are reported in Tables 3, 4. Among patients who did not receive PB, nulliparity was associated with severe pain (aOR 2.87, 95% CI 1.20–6.85; *p* = 0.018) and procedure suspension (aOR 3.07, 95% CI 1.27–7.39; *p* = 0.013), and cervical canal stenosis was associated with markedly increased odds of severe pain (aOR 23.17, 95% CI 10.44–51.40; *p* < 0.001) and procedure suspension (aOR 25.10, 95% CI 11.19–56.30; *p* < 0.001). Among patients who received PB, nulliparity was no longer associated with either outcome (severe pain aOR 0.88, 95% CI 0.54–1.43, *p* = 0.608; suspension aOR 1.05, 95% CI 0.60–1.83, *p* = 0.857), and the effect of cervical canal stenosis, although still significant, was substantially attenuated (severe pain aOR 2.68, 95% CI 1.57–4.58, *p* < 0.001; suspension aOR 3.70, 95% CI 2.07–6.62, *p* < 0.001).

**Table 3.** Influence of paracervical block on severe pain perception.

Severe pain perception and hysteroscopy							
Without paracervical block				With paracervical block			
	aOR	[95% IC]	p-value		aOR	[95% IC]	p-value
Demographic characteristics				Demographic characteristics			
Age	1.04	[0.99–1.09]	0.119	Age	0.97	[0.95–1.00]	0.064
No vaginal delivery	2.87	[1.20–6.85]	**0.018**	No Vaginal Delivery	0.88	[0.54–1.43]	0.608
Menopause	1.29	[0.39–4.28]	0.681	Menopause	0.91	[0.46–1.80]	0.788
Diseases	0.33	[0.13–0.83]	**0.018**	Diseases	0.77	[0.47–1.25]	0.296
Procedure				Procedure			
Cervical stenosis	23.17	[10.44–51.40]	**<0.001**	**Cervical Stenosis**	2.68	[1.57–4.58]	**<0.001**
Length	1.03	[0.98–1.07]	0.240	**Length**	1.04	[1.02–1.06]	**<0.001**

Significant results were evidenced using bold.

**Table 4.** Influence of paracervical block on suspension of procedure.

Suspension of procedure and hysteroscopy							
Without paracervical block				With paracervical block			
	aOR	[95% IC]	p-value		aOR	[95% IC]	p-value
Demographic characteristics				Demographic characteristics			
Age	1.01	[0.97–1.06]	0.591	Age	0.97	[0.94–1.00]	0.057
No vaginal delivery	3.07	[1.27–7.39]	**0.013**	No Vaginal Delivery	1.05	[0.60–1.83]	0.857
Menopause	2.94	[0.88–9.89]	0.081	Menopause	1.36	[0.64–2.91]	0.452
Diseases	0.36	[0.15–0.90]	**0.029**	Diseases	0.72	[0.47–1.25]	0.241
Procedure				Procedure			
Cervical stenosis	25.10	[11.19–56.30]	**<0.001**	Cervical stenosis	3.70	[2.07–6.62]	**<0.001**
Length	1	[0.94–1.05]	0.884	Length	1.01	[0.99–1.04]	0.294

Significant results were evidenced using bold.

A logistic model with an interaction term between PB and cervical canal stenosis formally tested whether this attenuation reflected an effect of PB rather than other observed covariates. The interaction-adjusted odds ratio for severe pain in patients with stenosis treated with PB versus without PB was 0.07 (95% CI 0.03–0.18, *p* < 0.001); for procedure suspension, 0.10 (95% CI 0.04–0.24, *p* < 0.001), corresponding to an approximately ten-fold reduction in the odds of both outcomes.

**Figure 4.**
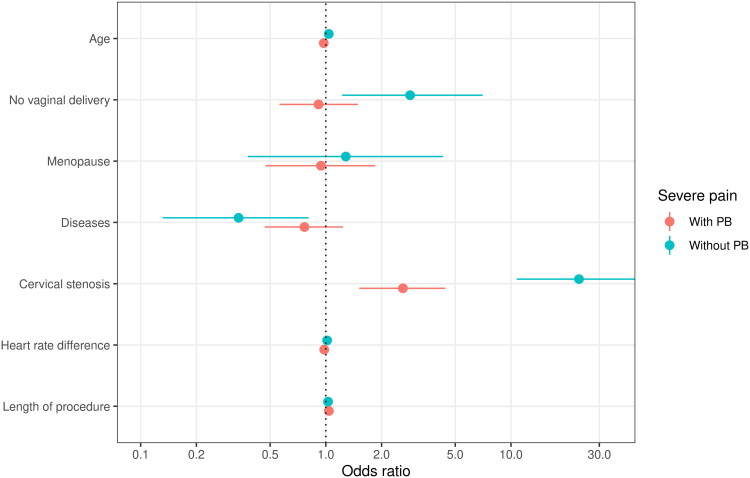
Influence of paracervical block on severe pain perception.

**Figure 5.**
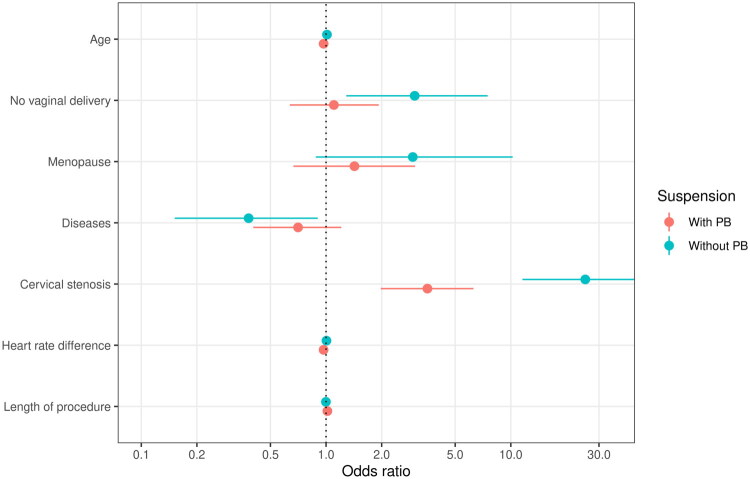
Influence of paracervical block on suspension of procedure.

PB was positively associated with longer procedure duration (aOR 1.04, 95% CI 1.02–1.06, *p* < 0.001), without a corresponding increase in the risk of suspension (aOR 1.01, 95% CI 0.99–1.04, *p* = 0.294) (Tables 3, 4; Figures 4, 5).

In our study we recorded PB correlated side effects only in 7 cases (0.53%), 5 of which with Vagal syndrome (0.38%), requiring drug therapy in only 2 cases (0.1%), and 3 cases of severe pain (0.22%). These complication rates are comparable to those described in literature [4,5].

## Discussion

Office hysteroscopy is the gold standard for the diagnosis and treatment of intracavitary uterine pathology [1] and has also proved valuable in the management of more complex conditions, such as cervical pregnancy [19]. The vaginoscopic approach has further consolidated its role in the outpatient setting by improving tolerability and procedural success [6]. Nevertheless, pain remains the leading cause of procedure suspension, with cervical manipulation representing the dominant mechanical trigger [4,16]. In this context, paracervical block—by directly inhibiting the nerve pathways responsible for cervical nociception—may serve as a complementary tool to VA, particularly in patients in whom the vaginoscopic technique alone is anticipated to be insufficient.

Pain perception during OH is, however, multifactorial. While cervical manipulation is the dominant mechanical contributor [16], the patient experience is also shaped by uterine distension, the type and duration of the operative steps, and a series of operator-related and contextual factors. Operator experience, a reassuring nursing team, “vocal local” techniques and the office environment further modulate pain perception, as do patient-related psychological factors such as pre-procedural anxiety, prior negative gynecological experiences and expectations [7]. Consistent with this multifactorial framework, Guraslan et al. [20], in a prospective cohort of 303 women undergoing diagnostic hysteroscopy with a mini-hysteroscope and without anesthesia, identified nulliparity (OR 4.6, 95% CI 1.7–13.2), postmenopausal status (OR 2.2, 95% CI 1.2–4.3), and unfavorable cervical canal features—particularly excessive cervical flexion and uterine retroversion (OR 4.1, 95% CI 2.0–8.5)—as independent risk factors for moderate-to-severe pain, even in experienced hands. These findings reinforce the view that pain during office hysteroscopy reflects an interaction of patient anatomy, cervical canal features, and patient-related factors that no single analgesic intervention can fully address. PB should therefore be understood as one modifiable factor among several, and the outcomes of OH depend on a coherent pain-management strategy of which PB is, in our practice, an important but not exclusive component.

The evidence on PB in OH is heterogeneous and of unequal methodological strength: older randomized trials are limited by small samples, restricted populations and routine use of speculum and tenaculum; systematic reviews stress the marked between-study heterogeneity and poor standardization of technique; and guideline recommendations have shifted from non-recommendation to the more nuanced position of the recent ACOG Clinical Consensus [11].

Among the available randomized trials, in the study of Cicinelli et al. PB was found effective in reducing pain perception and vasovagal symptoms, however, the study group was relatively small (36 cases vs 36 controls) and included only post-menopausal women [21]. In an RCT by Giorda et al. which evaluated 362 postmenopausal women who underwent diagnostic hysteroscopy with or without PB using 5 mm or 3.5 mm hysteroscopes, the perception of pain was reduced with the use of PB; however, the best results were obtained with smaller-caliber instruments [22].

More recently, the HYSPAIN trial [23], did not show a statistically significant reduction in VAS pain scores with paracervical mepivacaine 2% compared to saline placebo at any stage of the procedure. Two methodological aspects, however, limit direct comparability with our findings. First, the block was delivered with a spinal needle in the posterior fornix. Such an injection geometry is typically performed under speculum exposure, an inherently more invasive set-up than the pure vaginoscopic Vincenti’s-needle technique used in our cohort. Second, all HYSPAIN patients—in both arms—received standardized oral premedication (diazepam, ibuprofen and butylscopolamine) approximately one hour before the procedure. These differences in technique and background analgesia may contribute to reconciling the apparent discrepancy and suggest that any benefit of PB is unlikely to be uniform across all candidates for office hysteroscopy. Moving to the level of pooled evidence, the review by De Silva et al. in 2020 evaluated the effectiveness of local anesthesia during the OH in different sites (intracervical, paracervical, and intrauterine) and found it to be associated with a significant reduction in pain (SMD −0.57, 95% CI −0.79 to −0.34). However, it did not reduce vaso-vagal episodes. Besides, the authors conclude that further research is needed to understand the real benefits of pain control with local anesthesia and VA, as every method of analgesia was mostly performed by use of speculum and tenaculum [24]. A 2017 Cochrane review concluded that there was no good-quality evidence in terms of the safety or efficacy of the different types of local anesthesia in women undergoing OH [25]. Two further reviews highlighted the substantial heterogeneity between studies and the poor standardization of technique (different drugs, drug concentrations, with or without VA, with or without tenaculum), which limits the strength of pooled estimates and requires further confirmation [26,27].

Translating this evidence into practice, the main historical guidelines on outpatient hysteroscopy have advised against the routine use of PB [8–10]. However, the trials underpinning these recommendations administered PB almost exclusively with speculum and tenaculum [22,24–26,28–32], an inherently more invasive technique that is itself a source of pain and vasovagal stimulation, which limits the applicability of their findings to PB delivered through a purely vaginoscopic route. More recently, the ACOG Clinical Consensus No. 9 on Pain Management for In-Office Uterine and Cervical Procedures [11] has explicitly addressed hysteroscopy and recommends that clinicians proactively discuss pain-management options—including local anesthesia and paracervical block—with patients undergoing in-office uterine and cervical procedures. This marks a meaningful evolution of the normative framework, from a stance of non-recommendation to one of active counselling and shared decision-making. Nevertheless, the consensus does not specify how PB should be administered, and the empirical evidence available to inform such counselling still derives largely from speculum-and-tenaculum techniques. The present study addresses precisely this gap, by providing real-world data on PB performed *via* a vaginoscopic approach without speculum or tenaculum, using the “Vincenti’s” needle (Figure 2).

Our findings support previous literature on the causes and risk factors for severe pain during the procedure, highlighting nulliparity and cervical canal stenosis as the main reasons for procedure suspension and severe pain perception.

In the PB group, nulliparity was no longer associated with either severe pain or procedure suspension (Tables 3, 4). The passage through the fully innervated fibers of the uterine portio is a key determinant of pain perception during hysteroscopy, which is anatomically consistent with the role of a paracervical block in this setting. The very high odds of severe pain and procedure suspension observed with CC stenosis in patients not receiving PB (approximately 23- and 25-fold, respectively) were reduced to roughly 3-fold in patients who received PB (Tables 3, 4; Figures 4, 5), and the interaction-adjusted odds ratios indicated an approximately ten-fold reduction in both outcomes. These observations are consistent with a protective effect of PB in these subgroups but, given the retrospective design, must be interpreted with caution because of the inherent risk of confounding by indication.

Notably, although procedure duration is itself a risk factor for pain, patients in the PB group underwent longer procedures with a lower risk of suspension. This finding supports the feasibility of a “see and treat” approach, avoiding repeated procedures by allowing immediate treatment of pathology identified during the diagnostic step. By improving pain control during more demanding operative maneuvers, PB may help extend the applicability of this single-session model to a broader range of cases. This could change the experience perceived by women, who would feel less pain and obtain similar results without the need for hospitalization. Indeed, in our study, a significant number of patients successfully underwent operative procedures, such as polypectomy, myomectomy, and metroplasty, and most of them were in the PB group. Whether this reflects a true facilitating effect of PB or, conversely, a tendency to use PB precisely when more complex procedures were anticipated, cannot be fully disentangled from a retrospective design.

Stenosis of the CC was one of the most important factors for suspension of procedure and perception of severe pain; nevertheless, CC stenosis has not been universally defined. This condition is usually identified as the presence of an obliterated external or internal cervical ostium, or more broadly as a cervical canal that requires particular maneuvers for the introduction of the hysteroscope to access the uterine cavity [33].

**Figure 6.**
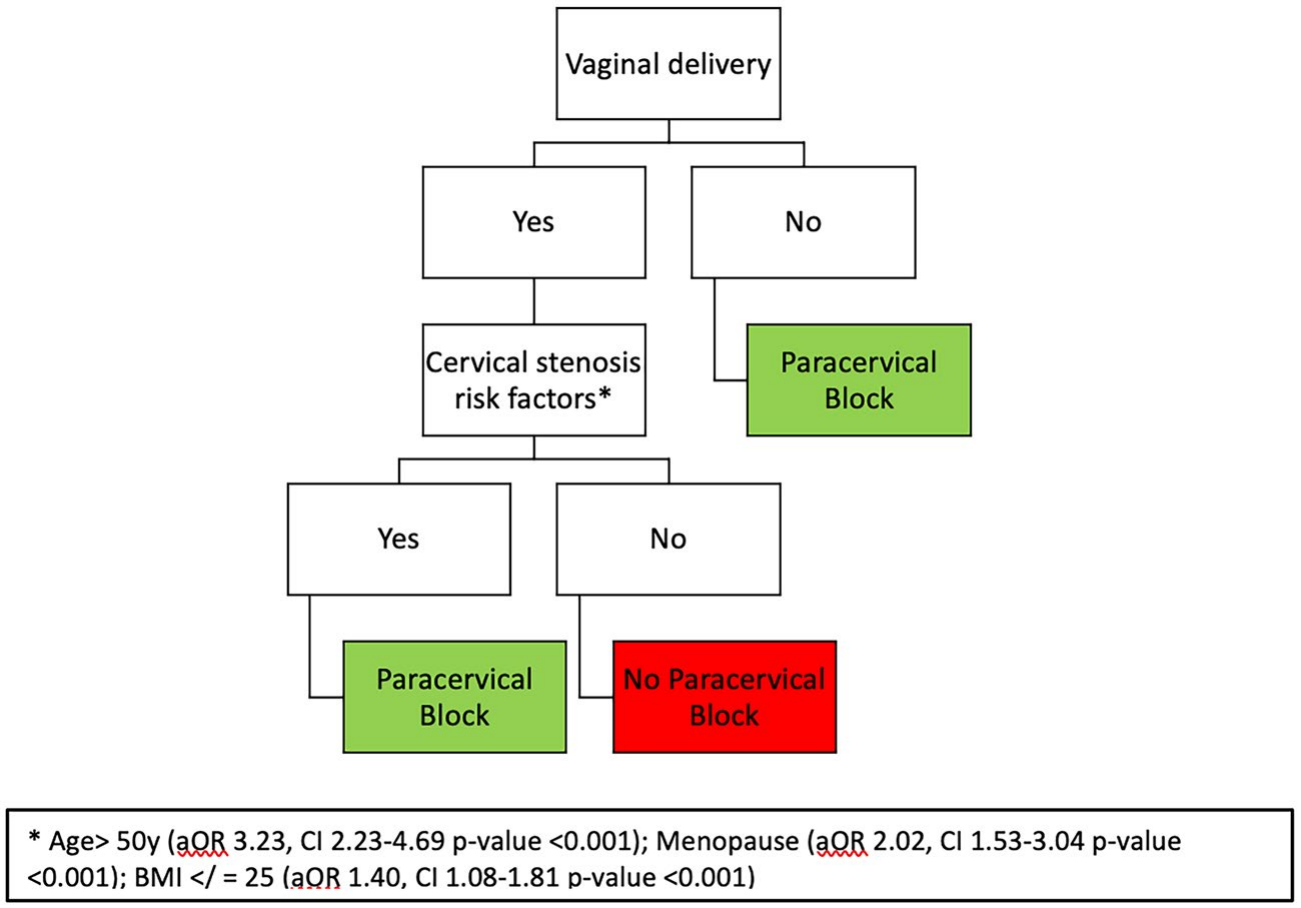
Execution algorithm of the paracervical block, during office hysteroscopy.

Because CC stenosis is often unpredictable, we identified five factors statistically associated with it in our population (Table 5) and derived a practice-based proposal to identify candidates who may benefit from PB (Figure 6). Nulliparity, per se, appeared in our cohort to be a relevant criterion for considering PB; in multiparous women, the predictable risk factors emerging from our population were age older than 50 years, menopausal status and a BMI lower than 25. The algorithm proposed is hypothesis-generating, not a clinical recommendation. Nonetheless, it formalizes the patient-selection process, coherently with the retrospective associations observed in this propensity-matched cohort. We offer it as a practice-based, single-center framework—a concrete starting point for prospective multicenter evaluation.

**Table 5.** Factors associated with cervical canal stenosis.

Stenosis	aOR	[95% IC]	*p*-value
Age > 50	3.23	[2.23–4.69]	**<0.001**
No vaginal delivery	2.02	[1.56–2.61]	**<0.001**
Menopause	2.16	[1.53–3.04]	**<0.001**
BMI ≤ 25	1.40	[1.08–1.81]	**0.010**

Significant results were evidenced using bold.

Contrary to what we expected, when analyzing the efficacy of PB considering the menopausal status alone, no statistically significant data was extracted. In the meantime, menopause has been found one of the principal risk factors for CC stenosis, consequently we can assume from our population that menopause should be considered as one of multiple variables to determine if the patient is eligible for a pre-hysteroscopy analgesia such as PB.

### Strengths and limitations

To the best of our knowledge, this is the only study to date evaluating PB in office hysteroscopy with a vaginoscopic approach, performed without speculum or tenaculum.

The strengths of the study include the routine use of VA, the large sample size, the detailed data collection, and the high standardized technique among the operators, despite their heterogeneity.

The limitations of our study include the retrospective design and data collection, which result in a lack of randomization. The use of PSM has partially exceeded such a limit. Confounding by indication remains an important interpretive caveat. PB administration was operator-dependent and likely preferential in patients anticipated to have a more difficult or painful procedure (e.g. suspected cervical stenosis, atrophic cervix, prior cervical surgery, marked anxiety). While PSM and multivariable adjustment balance measured covariates (age, parity, comorbidity), they cannot eliminate residual confounding from unmeasured factors—notably anticipated cervical access difficulty, the operator’s pre-procedural pain estimate, and patient anxiety—whose net direction on the estimated effect cannot be determined from the present data. The observed associations should therefore be regarded as clinically plausible but requiring confirmation in prospective randomized trials.

## Conclusions

In this large single-center retrospective cohort, PB administered through a vaginoscopic approach without speculum or tenaculum was associated with substantially lower odds of severe pain perception and procedure suspension in selected patients, namely nulliparous women and those with risk factors for cervical canal stenosis. These findings should be interpreted with caution, given the retrospective design and the non-randomized allocation of PB.

On this basis, our data support the consideration of PB as part of a structured pain-management strategy in patients with anticipated difficult cervical access, in line with the recent ACOG Clinical Consensus on pain management for in-office uterine and cervical procedures. The proposed practice-based algorithm (Figure 6) should likewise be regarded as hypothesis-generating. Prospective randomized studies are needed to confirm the role of PB in OH performed with VA, without speculum and tenaculum, and to better define the patient subgroups in whom its benefit clearly outweighs the additional maneuver of the injection.

## Data Availability

Data regarding any of the subjects in the study has not been previously published. Data will be made available to the editors of the journal for review or query upon request.
